# Development of the COVID-19 Perinatal Perception Questionnaire (COVID19-PPQ)

**DOI:** 10.1007/s10862-021-09900-4

**Published:** 2021-07-17

**Authors:** Lianne P. Hulsbosch, Myrthe G. B. M. Boekhorst, Lotte Muskens, Eva S. Potharst, Ivan Nyklíček, Victor J. M. Pop

**Affiliations:** 1 Center of Research in Psychological and Somatic Disorders (CoRPS), Department of Medical and Clinical Psychology, Tilburg University, P.O. BOX 90153, 5000 LE Tilburg, the Netherlands; 2 UvA Minds, academic outpatient (child and adolescent) treatment center of the University of Amsterdam, Amsterdam, the Netherlands; 3 Research Institute of Child Development and Education, University of Amsterdam, Amsterdam, the Netherlands

**Keywords:** COVID-19 pandemic, Perception, Perinatal, Validation

## Abstract

The COVID-19 pandemic affects the mental health status of perinatal women, which makes it important to gain insight into and to effectively measure specific stressors of the COVID-19 pandemic. Therefore, we aimed to develop a COVID-19 Perinatal Perception Questionnaire (COVID19-PPQ). In-depth interviews were conducted during the first national lockdown period with pregnant women, new mothers and perinatal healthcare professionals, resulting in (a) a 27-item pregnancy and (b) a 21-item postpartum scale. Explorative factor analyses (EFA) in sample Ia (N = 154) and Ib (N = 90), and confirmatory factor analyses (CFA) in sample IIa (N = 113) and IIb (N = 81) were conducted to test the psychometric properties of both scales. For the pregnancy scale, EFA suggested a three-factor solution (risk of infection, contact, future), which was confirmed by CFA and resulted in a final nine-item scale. For the postpartum scale, a three-factor solution (first postpartum week, COVID-19 measures, fear for infection) was suggested by EFA and confirmed by CFA, resulting in a final ten-item scale. Symptoms of depression and pregnancy-specific distress were significantly correlated with the pregnancy (sub)scale(s), while symptoms of postpartum depression and anxiety showed significant correlations with the COVID-19 measures and fear for infection subscale. The COVID19-PPQ seems to be a valid instrument for assessment of perinatal COVID-19-related stress perception, showing adequate psychometric properties for both the pregnancy and postpartum scale. Future research should examine the use of this instrument in clinical practice during new episodes of the COVID-19 pandemic.

## Introduction

The COVID-19 pandemic and lockdown have a major impact on the psychological and social wellbeing of individuals (Rajkumar, 2020). Especially women in the perinatal period may have been affected, since these women are more vulnerable for environmental stressors (Bales et al., 2015) and more often experience elevated levels of distress (Dennis et al., 2017; Woody et al., 2017), which is a risk factor for maternal mental health problems (Dayan et al., 2010). During the COVID-19 pandemic, pregnant and postpartum women showed elevated levels of depressive and anxiety symptoms, compared to similar pre-pandemic groups (Ceulemans et al., 2020; Davenport et al., 2020; Lebel et al., 2020; Wu et al., 2020). Moreover, pregnant women showed an increase in pregnancy-specific distress symptoms (Boekhorst et al., 2020a, b). Pregnant women expressed more concerns about the health of their unborn baby as well as more fear, sadness and uncertainty regarding their childbirth during the COVID-19 pandemic (Ravaldi et al., 2020). Another study showed that almost half of the participating pregnant women reported high anxiety for vertical transmission of COVID-19 (Saccone et al., 2020). Moreover, the COVID-19 pandemic was associated with a decrease in mother-infant bonding at one month postpartum (Suzuki, 2020).

It seems likely that the lockdown restrictions regarding pregnancy care, work and social activities have affected women in the perinatal period in various other ways as well. It is important to gain insight into the specific aspects of stressors of the COVID-19 pandemic affecting the mental health status in the perinatal period, and to measure these stressors effectively. Therefore, the aim of the current study was to develop a COVID-19 Perinatal Perception Questionnaire (COVID19-PPQ), using input by in-depth interviews of pregnant women, women who gave birth recently and perinatal healthcare professionals.

## Method

### Participants and Procedure

The current study is part of the Brabant Study, a large longitudinal cohort study following women from 12 weeks of pregnancy until eight to ten weeks postpartum (see Meems et al. (2020) for the design of the study). The study was approved by the Medical Ethics Committee at the Máxima Medical Centre Veldhoven (L64091.015.17). Written informed consent was obtained from all participants.

In the current study, specific aspects of stressors of the COVID-19 pandemic in the perinatal period, were assessed by telephone interviews with a selection of participants from the Brabant Study. Interviews were conducted during the first national lockdown period with nine primiparous and ten multiparous pregnant women between 12 and 28 weeks of pregnancy, and seven primiparous and eight multiparous women who recently gave birth. These interviews took place from 21 April until 20 May 2020. Moreover, telephone interviews were conducted with healthcare professionals (five community midwifes, three clinical midwives, five obstetricians and three maternity nurses) from 14 May until 27 May 2020. All interviews were conducted by researchers from the university. The interviews were recorded and transcribed by research assistants. Subsequently, two researchers independently identified important themes from the interviews with the Brabant Study participants, and two other researchers identified themes from the interviews with the healthcare professionals. Thereafter, possible candidate items for the questionnaire were formulated per theme and discussed by these four researchers and supervised and confirmed by a fifth researcher. Double and less relevant items were eliminated until a consensus was reached. This resulted in 27 candidate items for the pregnancy scale and 21 candidate items for the postpartum scale, assessing the perinatal COVID-19-related stress perception. Items were formatted on a four-point Likert scale, ranging from 1 (‘completely disagree’) to 4 (‘fully agree’).

In July and August 2020, 154 pregnant women (sample Ia) and 90 postpartum women (sample Ib) completed the 27-item pregnancy scale and the 21-item postpartum scale, respectively. Data of sample I were used to conduct an exploratory factor analysis (EFA) and reliability analysis. This resulted in a more refined version of the COVID19-PPQ. This refined version was filled out by 113 pregnant women (sample IIa) and 81 postpartum women (sample IIb) in September and October 2020. Data of this sample II were used to perform a confirmatory factor analysis (CFA). Except for gestational age in sample Ia and IIa, the women in sample I and II had similar characteristics and therefore the data of both samples was subsequently taken together to examine the concurrent validity.

### Measures

#### Depressive Symptoms

Depressive symptoms during pregnancy (12, 20, 28 weeks) and postpartum (8–10 weeks) were assessed using the Dutch version of the ten-item Edinburgh (Postpartum) Depression Scale (E(P)DS) (Bergink et al., 2011; Cox et al., 1987; Pop et al., 1992). The total score ranges from 0 to 30 and a higher score indicates more depressive symptoms. The E(P)DS has been shown to be a valid and reliable instrument to measure depressive symptoms in each trimester of pregnancy (Bergink et al., 2011) and postpartum (Pop et al., 1992). In the current study, the Cronbach’s alpha’s were .85, .84 and .86 per trimester, respectively and .83 postpartum.

#### Pregnancy-Specific Distress

Measurement of pregnancy-specific distress symptoms was carried out using the negative affect subscale (TPDS-NA, ten-item adapted version) of the Tilburg Pregnancy Distress Scale (TPDS) (Pop et al., 2011). Total scores range from 0 to 30 and higher scores indicate greater levels of pregnancy-specific distress. The TPDS-NA shows good psychometric properties in each trimester of pregnancy (Boekhorst et al., 2020a, b), and the internal consistency and structural validity have been evaluated as excellent (Evans et al., 2015). In the current study, the Cronbach’s alpha’s were .76, .81 and .76, per trimester, respectively.

#### Postpartum Anxiety Symptoms

The ten-item anxiety subscale of the Dutch version of the Symptom Checklist (SCL-90) was used to assess anxiety symptoms postpartum (8–10 weeks) (Arrindell & Ettema, 1981). Total scores range from 0 to 40 and higher scores reflect more anxiety symptoms. The SCL-90 has shown to have good reliability and validity (Arrindell & Ettema, 2003). The Cronbach’s alpha in the current study was .86.

#### Descriptive Characteristics

Demographic, psychological and obstetric parameters were obtained at baseline in the first trimester of pregnancy, such as age, high level of education (Bachelor’s or Master’s degree), paid job, living with a partner, previous diagnosis of depression and/or anxiety disorder, parity, previous miscarriage, problems with previous pregnancy and/or delivery.

### Statistical Methods

Statistical analyses were performed using the Statistical Package for Social Sciences (SPSS version 24, IBM, Chicago IL, USA). CFA was completed using AMOS (version 24, IBM, Chicago, IL, USA). EFA was performed on the 27-item pregnancy scale and the 21-item postpartum scale in sample I. We used a principal component analysis and scree plot to select factors for retention. Item factor loadings > .40 were regarded to be important. When items loaded on more than one dimension, we retained the item when the difference was at least .20, unless face validity and reliability suggested otherwise. Internal consistency analyses were conducted using Cronbach’s alpha for the total pregnancy and postpartum scale and possible subscales derived from factor analysis.

Thereafter, CFA was performed in sample II on the remaining items from the refined version of the pregnancy and postpartum scale of the COVID19-PPQ. CFA was used to test the model fit of the factor structures found with EFA, assessing the comparative fit index (CFI), normed fit index (NFI), Tucker-Lewis Index (TLI), and the root mean square error of approximation (RMSEA) with lower bound. Adequate model fit can be assumed with a CFI ≥ .80, NFI ≥ .80, TLI ≥ .80, and RMSEA ≤ .08 for adequate and RMSEA ≤ .05 for excellent fit with an appropriate lower bound set at .04 (Browne & Cudeck, 1992; Hu & Bentler, 1998).

To assess concurrent validity of the pregnancy and postpartum scale of the COVID19-PPQ, we correlated the pregnancy scale with the EDS and TPDS-NA in each trimester of pregnancy and the postpartum scale with the EPDS and SCL-90 (Pearson’s *r* correlations, two-tailed).

## Results

### Factor Analyses

#### COVID19-PPQ: Pregnancy Scale

The women participating in samples Ia and IIa had similar characteristics except gestational age (Table 1a). All item scores were normally distributed in sample Ia. See Table 2 for an overview of items. Based on face validity, items 16, 19 and 21 were eliminated. The Kaiser–Meyer–Olkin index was > .60 (.73) with a significant Bartlett’s test of sphericity (*p* < .001). A three-factor solution was suggested in the scree plot, with a ‘risk of infection’ factor, ‘contact’ factor and ‘future’ factor, explaining 38.6% of the variance (Table 2). A varimax rotation was used since the component correlations between the three factors were < .30 (.28) with direct oblimin rotation. Four items (1, 6, 25 and 26) did not have a loading above .40 and five items (4, 7, 9, 18 and 20) loaded on both factors with a difference smaller than .20 and were therefore omitted. Items 11 and 15 were retained because of face validity and reliability, while item 8 was omitted because of face validity. Reliability analyses resulted in a Cronbach’s alpha of .74 for the four-item risk of infection subscale and a Cronbach’s alpha of .72 for the six-item contact subscale. The four-item future subscale had a Cronbach’s alpha of .70, which increased to .71 after deletion of item 24. The total 13-item pregnancy scale of the COVID19-PPQ had a Cronbach’s alpha of .76.

**Table 1:** Characteristics of two samples of pregnant women (N = 267) and two samples of postpartum women (N = 171)

**a. Pregnant women**								
	Sample Ia (N = 154)				Sample IIa (N = 113)			
*Demographics*	N	%	Mean (SD)	Range	N	%	Mean (SD)	Range
Age			30.7 (3.2)	19–40			31.0 (3.7)	20–41
High level of education	111	73.5			76	68.5		
Paid job	146	96.7			110	99.1		
Living with partner	147	97.4			110	99.1		
*Pregnancy related*								
Gestational age			35.1 (5.1)	24–43			22.7 (5.2)	13–33
Multiparity	66	43.7			44	39.6		
Unplanned pregnancy	13	8.6			9	8.1		
Previous miscarriage	39	25.8			30	27.0		
Problems with previous pregnancy	29	18.8			14	12.4		
Problems with previous delivery	43	28.5			25	22.5		
*Psychological features*								
Previous diagnosis of depression	22	14.6			17	15.3		
Previous diagnosis of anxiety disorder	10	6.6			10	9.0		
EDS 1^st^ trimester			4.9 (4.7)	0–23			4.5 (4.3)	0–21
EDS 2^nd^ trimester			5.1 (4.4)	0–21			4.7 (4.1)	0–17
EDS 3^rd^ trimester			4.9 (4.7)	0–25			6.0 (5.1)	0–22
TPDS-NA 1^st^ trimester			6.1 (4.0)	0–18			5.6 (3.2)	0–14
TPDS-NA 2^nd^ trimester			5.5 (4.3)	0–18			5.2 (3.7)	0–15
TPDS-NA 3^rd^ trimester			11.1 (7.6)	0–30			12.1 (7.7)	0–30

**b. Postpartum women**								
	Sample Ib (N = 90)				Sample IIb (N = 81)			
*Demographics*	N	%	Mean (SD)	Range	N	%	Mean (SD)	Range
Age			31.1 (3.4)	22–39			30.7 (3.2)	24–38
High level of education	63	71.6			62	76.5		
Paid job	83	94.3			80	98.8		
Living with partner	87	98.9			79	97.5		
*Pregnancy related*								
Multiparity	46	52.3			40	49.4		
Unplanned pregnancy	6	6.8			6	7.4		
Previous miscarriage	22	25.0			22	27.2		
Problems with previous pregnancy	23	25.6			20	24.7		
Problems with previous delivery	30	34.1			26	31.1		
*Obstetric features*								
Gestational age at delivery (weeks)			39.6 (1.4)	35–42			39.4 (1.7)	29–41
Premature birth (< 37 weeks)	4	4.4			4	5.0		
*Psychological features*								
Previous diagnosis of depression	10	11.4			11	13.6		
Previous diagnosis of anxiety disorder	6	6.8			8	9.9		
EPDS			4.4 (3.4)	0–15			4.6 (4.0)	0–19
SCL-90			2.4 (2.7)	0–13			2.5 (3.4)	0–18

SD, standard deviation; High level of education, Bachelor’s or Master’s degree; E(P)DS, Edinburgh (Postpartum) Depression Scale; TPDS-NA, Negative Affect subscale of the Tilburg Pregnancy Distress Scale; SCL-90, Anxiety subscale of the Symptom Checklist

**Table 2:** Three-factor solution from explorative factor analysis (EFA) with varimax rotation in 154 (sample Ia) pregnant women who completed the 27-item pregnancy scale of the COVID19-PPQ

	Factor I *Risk of infection*	Factor II *Contact*	Factor III *Future*
Eigenvalue	4.9	2.3	2.1
Percentage of variance explained	20.2	9.7	8.7
1. I trusted my obstetrician’s knowledge about Corona	.32		.29
**2. I missed the personal contact with my obstetrician**		**.47**	
**3. It bothered me that scheduled ultrasounds were not able to continue**		**.64**	
4. I understood that Corona measures had to be taken		.50	.37
**5. I was upset that my partner was not allowed to be present during the ultrasounds**		**.57**	
6. I plan to deliver my baby at home because of Corona			.25
7. I was afraid that my partner would not be allowed to be present during the delivery	.33		.42
8. I was upset that perinatal classes were canceled	.57		
9. I thought the lockdown measures were too strict	-.24	.45	.34
**10. I did not want to go to the hospital for a consultation because of the risk for infection**	**.51**		.21
**11. Because of the Corona measures, I did not enjoy my pregnancy as much**	.37	**.54**	.26
**12. I was worried that a possible Corona infection would impact my baby**	**.71**		
**13. Being pregnant made me feel more at risk for the Coronavirus**	**.78**		
**14. I strictly adhered to the Corona measures because I was pregnant**	**.73**		
**15. I was upset that I could share less with family and friends during my pregnancy**	.43	**.54**	
16. The people in my environment were considerate of the fact that I was pregnant			
**17. I was worried that I could not have visitors after the baby was born**		**.64**	
18. Because of the lockdown I could share my experiences about my pregnancy with fewer people than I wanted to	.47	.48	
19. Because my partner was more at home during the lockdown my pregnancy was really a shared experience			
20. Because of the lockdown I felt more peace (fewer social activities)		.53	.33
21. During the lockdown my partner and I were living more on top of one another			
**22. Because of Corona I am more worried about my financial future**			**.76**
**23. I am worried that Corona will affect my job**			**.74**
**24. I am confident about the period after my pregnancy**	.21	.22	**.50**
25. Because of the lockdown I enjoy my job less	.28		.23
26. I have experienced the changes in my work activities as positive		.31	
**27. I am worried that Corona will affect my partner’s job**			**.61**

Items 16, 19 and 21 were eliminated based on face validity. To retain items (**bold**, n = 9) a cut-off score of item loading of .40 was used and a minimum difference of .20 if an item had two loadings, unless face validity and reliability suggested otherwise. Total explained variance is 38.6%

When we performed a CFA on the pregnancy scale in the second pregnancy sample (IIa) with the items and dimensions of the EFA of the first sample (Ia), we found a poor model fit (CFI = .75, NFI = .68, TLI = .69, RMSEA = .13, lower bound = .11). The standardized residual covariance matrix clearly showed that items 2, 11, 14 and 15 showed relationships that were not well explained by the model and were therefore removed. When we repeated the CFA without these items we found an adequate model fit (CFI = .95, NFI = .88, TLI = .92, RMSEA = .07, lower bound = .03). Then, EFA with varimax rotation was performed on the nine-item pregnancy scale in sample IIa for confirmation, which resulted in a three factor structure with a total explained variance of 67.5%. The three-item risk of infection subscale had a Cronbach’s alpha of .74, while the three-item contact and three-item future subscales both had a Cronbach’s alpha of .71. The total nine-item pregnancy scale showed a Cronbach’s alpha of .71. The items were recoded from 1–4 to 0–3, with total scores ranging from 0 to 27. Higher scores indicated a more negative COVID-19-related stress perception during pregnancy.

#### COVID19-PPQ: Postpartum Scale

Women in samples Ib and IIb had similar characteristics (Table 1b). All item scores were normally distributed in sample Ib. A Kaiser–Meyer–Olkin index greater than .60 (.76) was found and the Bartlett’s test of sphericity value was significant (*p* < .001). The scree plot suggested a three-factor solution with 47.3% total explained variance, with a ‘first postpartum week’ factor, ‘COVID-19 measures’ factor and ‘fear for infection’ factor (Table 3). The component correlations between the three factors were smaller than .30 (.18) with direct oblimin rotation and therefore varimax rotation was used. Three items (2, 11 and 15) did not have a loading > .40 and four items (7, 16, 19 and 21) loaded on both factors with a difference < .20 and were therefore eliminated. Reliability analyses showed a Cronbach’s alpha of .77 for the six-item first postpartum week subscale, which increased to .81 after omission of item 1. A Cronbach’s alpha of .77 was found for the five-item COVID-19 measures subscale and a Cronbach’s alpha of .66 for the three-item fear for infection subscale, which increased to .84 after elimination of item 20. The total 12-item postpartum scale of the COVID19-PPQ had a Cronbach’s alpha of .78.

**Table 3:** Three-factor solution from explorative factor analysis (EFA) with varimax rotation in 90 (sample Ib) postpartum women who completed the 21-item postpartum scale of the COVID19-PPQ

	Factor I *First postpartum week*	Factor II *COVID-19 measures*	Factor III *Fear for infection*
Eigenvalue	5.4	2.7	1.8
Percentage of variance explained	25.8	12.8	8.7
**1. I was upset that only my partner could be present during my delivery**	**.40**		
2. My delivery was different from what I had expected because of Corona	.30	.35	.36
**3. I was afraid to be infected with the Coronavirus during the delivery/first postpartum week**			**.81**
**4. I was afraid that my baby would be infected with the Coronavirus during the delivery/first postpartum week**			**.83**
**5. I received conflicting information during my delivery**		**.66**	
**6. I trusted that the midwife/obstetrician would strictly adhere to the Corona measures during my delivery**		**.69**	
7. The contact with healthcare professionals was less personal during my delivery because of Corona	.21	.43	.34
**8. I had an understanding for the Corona measures during my delivery**	.26	**.69**	
**9. The Corona measures that were taken during my delivery were clear**		**.70**	
**10. I felt very lonely during my delivery because of the Corona measures**	.38	**.66**	.26
11. I missed my midwife providing my aftercare during the first postpartum week		.36	.31
**12. It felt unfortunate that I could have fewer visitors after my delivery**	**.65**		
**13. I enjoyed the peace and quiet during the first postpartum week**	**.83**		
**14. There was more time and attention to get breastfeeding started**	**.70**		
15. The maternity nurse sufficiently adhered to the Corona measures	.24	.31	
16. Because of the Corona measures I enjoyed the first postpartum week less	.44	.54	
**17. I feel that the first postpartum week was nice and peaceful because of the Corona measures**	**.79**	.28	
**18. Because of the Corona measures I felt more connected to my (new) family**	**.72**		
19. Apart from our family I did not allow anyone to hold the baby	-.36	.27	.52
**20. During the period after my delivery I hardly went outside**			**.46**
21. Because of the Corona measures I felt very lonely during the period after my delivery	.35	.38	.46

To retain items (**bold**, n = 9) a cut-off score of item loading of .40 was used and a minimum difference of .20 if an item had two loadings, unless face validity and reliability suggested otherwise. Total explained variance is 47.3%

Several women that completed the first version of the postpartum scale mentioned that they missed a question on fear for infection of their partner. Therefore, we included an extra question in version two of the scale: “I was afraid that my partner would be infected with the Coronavirus during the delivery/first postpartum week”. CFA was then conducted on the 13-item second version of the postpartum scale in sample IIb and resulted in an inadequate model fit (CFI = .77, NFI = .72, TLI = .75, RMSEA = .09, lower bound = .06). The standardized residual covariance matrix clearly showed that items 5, 10 and 12 showed relationships that were not well explained by the model. These items were subsequently removed and when we repeated the CFA on the remaining ten items we found an excellent model fit (CFI = .98, NFI = .92, TLI = .97, RMSEA = .03, lower bound = .01). We repeated EFA with varimax rotation on the ten-item postpartum scale in sample IIb, for confirmation. This resulted in a three factor structure, explaining 72.1% of the variance. Reliability analyses showed a Cronbach’s alpha of .83 for the postpartum subscale, .77 for the COVID-19 measures subscale, .87 for the fear for infection subscale, and .64 for the total ten-item postpartum scale. The items were recoded from 1–4 to 0–3, and the items of the first postpartum week and COVID-19 measures subscale were reverse coded. Total scores ranged from 0 to 30 with higher scores indicating a more negative COVID-19-related stress perception postpartum. Table 4 shows the pregnancy and postpartum scale of the COVID19-PPQ.

**Table 4:** The COVID-19 Perinatal Perception Questionnaire (COVID19-PPQ)

***Pregnancy scale***	Completely disagree (1)	Disagree (2)	Agree (3)	Fully agree (4)
1. It bothered me that scheduled ultrasounds were not able to continue	□	□	□	□
2. I was upset that my partner was not allowed to be present during the ultrasounds	□	□	□	□
3. I did not want to go to the hospital for a consultation because of the risk for infection	□	□	□	□
4. I was worried that a possible Corona infection would impact my baby	□	□	□	□
5. Being pregnant made me feel more at risk for the Coronavirus	□	□	□	□
6. I was worried that I could not have visitors after the baby was born	□	□	□	□
7. Because of Corona I am more worried about my financial future	□	□	□	□
8. I am worried that Corona will affect my job	□	□	□	□
9. I am worried that Corona will affect my partner’s job	□	□	□	□

***Postpartum scale***	Completely disagree (1)	Disagree (2)	Agree (3)	Fully agree (4)
1. I was afraid to be infected with the Coronavirus during the delivery/first postpartum week	□	□	□	□
2. I was afraid that my baby would be infected with the Coronavirus during the delivery/first postpartum week	□	□	□	□
3. I trusted that the midwife/obstetrician would strictly adhere to the Corona measures during my delivery	□	□	□	□
4. I had an understanding for the Corona measures during my delivery	□	□	□	□
5. The Corona measures that were taken during my delivery were clear	□	□	□	□
6. I enjoyed the peace and quiet during the first postpartum week	□	□	□	□
7. There was more time and attention to get breastfeeding started	□	□	□	□
8. I feel that the first postpartum week was nice and peaceful because of the Corona measures	□	□	□	□
9. Because of the Corona measures I felt more connected to my (new) family	□	□	□	□
10. I was afraid that my partner would be infected with the Coronavirus during the delivery/first postpartum week	□	□	□	□

***Pregnancy scale***. Subscale risk of infection = item 3, 4, 5. Subscale contact = item 1, 2, 6. Subscale future = item 7, 8, 9. All items recoded from 1–4 to 0–3. ***Postpartum scale***. Subscale first postpartum week = item 6, 7, 8, 9. Subscale COVID-19 measures = item 3, 4, 5. Subscale fear for infection = item 1, 2, 10. Items 1, 2, 10 recoded from 1–4 to 0–3. Items 3, 4, 5, 6, 7, 8, 9 recoded from 1–4 to 3–0

### Concurrent Validity Analyses

Sample I and II were combined for concurrent validity with the E(P)DS, TPDS-NA and SCL-90. We used multiple imputation with ten iterations on the missing values in sample IIa, corresponding to the item that was included in the postpartum scale after EFA. The final ten items of the postpartum scale were included in the imputation process. A normal distribution was shown for all items in the pregnancy and postpartum scale. The Pearson correlations between the pregnancy scale and the EDS and TPDS-NA in each trimester of pregnancy are shown in Table 5a. In sum, the EDS correlated significantly with most pregnancy (sub)scale(s) with small effect size, while the coefficients with the TPDS-NA showed small to medium effect sizes. Table 5b shows that the postpartum scale and the COVID-19 measures and fear for infection subscale were significantly associated with the EPDS, as well as with the SCL-90, all with small effect sizes.

**Table 5:** Correlation coefficients between the pregnancy scale of the COVID19-PPQ and symptoms of depression and pregnancy-specific distress at each trimester of pregnancy (N = 267) and between the postpartum scale of the COVID19-PPQ and symptoms of postpartum depression and anxiety (N = 171)

**a. Pregnancy scale**	1	2	3	4	5	6	7	8	9	10	Mean (SD)	Range
1. Pregnancy scale	1	.69***	.77***	.58***	.21**	.18**	.24**	.37***	.32***	.37***	11.7 (4.6)	0–24
2. Pregnancy scale: Risk of infection	-	1	.31***	.15*	.14*	.17**	.19**	.30***	.30***	.30***	4.1 (2.1)	0–9
3. Pregnancy scale: Contact	-	-	1	.14*	.16*	.06	.10	.25***	.16*	.24**	5.1 (2.6)	0–9
4. Pregnancy scale: Future	-	-	-	1	.12^†^	.16*	.19**	.22***	.19**	.18*	2.5 (2.0)	0–9
5. EDS 1^st^ trimester	-	-	-	-	1	.61***	.64***	.54***	.38***	.37***	4.8 (4.5)	0–23
6. EDS 2^nd^ trimester	-	-	-	-	-	1	.66***	.46***	.47***	.36***	4.9 (4.3)	0–21
7. EDS 3^rd^ trimester	-	-	-	-	-	-	1	.40***	.38***	.37***	5.1 (4.8)	0–25
8. TDPS-NA 1^st^ trimester	-	-	-	-	-	-	-	1	.70***	.65***	5.9 (3.7)	0–18
9. TDPS-NA 2^nd^ trimester	-	-	-	-	-	-	-	-	1	.62***	5.1 (4.1)	0–18
10. TDPS-NA 3^rd^ trimester	-	-	-	-	-	-	-	-	-	1	11.3 (7.6)	0–30

**b. Postpartum scale**	1	2	3	4	5	6	Mean (SD)	Range				
1. Postpartum scale	1	.77***	.51***	.59***	.18*	.18*	9.9 (3.9)	1–20				
2. Postpartum scale: First postpartum week	-	1	.22***	-.11	-.01	-.08	3.9 (2.6)	0–12				
3. Postpartum scale: COVID-19 measures	-	-	1	.05	.17*	.17*	2.3 (1.7)	0–9				
4. Postpartum scale: Fear for infection	-	-	-	1	.20*	.28**	3.7 (2.2)	0–9				
5. EPDS	-	-	-	-	1	.64***	4.5 (3.7)	0–19				
6. SCL-90	-	-	-	-	-	1	2.5 (3.0)	0–18				

COVID19-PPQ, COVID-19 Perinatal Perception Questionnaire; E(P)DS, Edinburgh (Postpartum) Depression Scale; TPDS-NA, Negative Affect subscale of the Tilburg Pregnancy Distress Scale; SCL-90, Anxiety subscale of the Symptom Checklist

^†^*p* < .10, *p < .05, **p < .01, ***p < .001 (two-tailed)

## Discussion

In the current study, the COVID19-PPQ was developed during the first national lockdown period based on input from pregnant women, new mothers and perinatal healthcare professionals. The questionnaire consists of an (a) pregnancy and (b) postpartum scale. We found adequate psychometric properties for both scales: a three-factor structure for the pregnancy scale with appropriate reliability and adequate model fit, and a three-factor structure for the postpartum scale with appropriate reliability and excellent model fit. It must be noted that the Cronbach’s alpha of the total postpartum scale was .64, while a Cronbach’s alpha of > .70 has been recommended (Kline, 1993). This could be due to the reverse coded items of the first postpartum week and COVID-19 measures subscale, especially since the reliability scores of the three subscales were all > .77. This could imply that using the subscale scores instead of the total postpartum scale score may be preferable.

The pregnancy scale consists of three subscales. Items of the risk of infection subscale refer to worries about infection and risk of COVID-19 during pregnancy. The contact subscale contains items involving concerns about cancellations of ultrasounds, ultrasounds without partner and lack of visits from family and friends during the first postpartum week. Items of the future subscale entail worries about finances and work. All subscales appear to have a concern or worry component. Indeed, the total pregnancy scale and its subscales were significantly correlated with pregnancy specific distress symptoms with medium effects size. In addition, there was a significant correlation between the pregnancy (sub)scale(s) and symptoms of depression, but with small effect size.

The postpartum scale contains three subscales, with items of the first postpartum week subscale referring to perception of COVID-19-related changes in the first postpartum week, especially due to fewer visits from family and friends. The COVID-19 measures subscale contains items regarding the perception of the measures and guidelines during delivery. Items in the fear for infection subscale include worries to get infected (self, baby or partner) with COVID-19 during delivery or in the first postpartum week. The COVID-19 measures and fear for infection subscale showed a significant correlation with symptoms of postpartum depression and anxiety with small effect size, and the first postpartum week subscale showed no significant associations. This could imply that, in contrast to pregnant women, there were certain stressors of the COVID-19 pandemic in postpartum women that did not have an association with elevated levels of depression and anxiety. Another explanation could be that there were lower levels of stressors postpartum. Moreover, when looking at effect sizes, perinatal stressors of the COVID-19 pandemic seemed to have a greater association with distress symptoms in pregnant women than in postpartum women.

The concept of the COVID19-PPQ could be clinically relevant in measuring specific COVID-19 stressors that could be related to increased depression and anxiety symptoms in perinatal women in times of COVID-19 (Ceulemans et al., 2020; Davenport et al., 2020; Lebel et al., 2020; Wu et al., 2020). Knowing on which subscale(s) of the pregnancy and postpartum scale perinatal women show higher levels of stressors, could help healthcare professionals in offering proper support to perinatal women during the COVID-19 pandemic. Moreover, the stressors that were identified in the current study could be valuable in developing interventions for perinatal women in the current and future pandemic episodes. A meta-analysis stressed the need for such interventions based on evidence from past epidemics and pandemics (Shorey & Chan, 2020).

The development of the COVID19-PPQ was based on in-depth interviews with perinatal women and healthcare professionals during the first lockdown period, which is a major strength of the current study. A limitation is that compared to the national population, all participants were Dutch, more often highly educated and living with a partner, which could limit generalization of our findings. Therefore, the psychometric properties of the COVID19-PPQ should be re-evaluated in samples with more diversity in ethnicity, education level and type of household.

In conclusion, the COVID19-PPQ seems to be a user-friendly and valid instrument for assessment of perinatal COVID-19-related stress perception, with adequate psychometric properties for both the pregnancy and postpartum scale. Future research should examine the use of this instrument in clinical practice during new episodes of the COVID-19 pandemic.

## Data Availability

The datasets generated and analyzed during the current study are available from the corresponding author on reasonable request.

## Declarations

### Experiment participants

All procedures conducted in the current study were approved by the local Medical Ethics Committee.
